# Challenges in the assessment of epithelial dysplasia in oral lichen planus and oral lichenoid lesion: Inter and intra-observer variability of the WHO criteria and binary system

**DOI:** 10.4317/jced.61073

**Published:** 2024-01-01

**Authors:** Letícia-Côgo Marques, Pâmella-de Pinho Montovani, Lúcio-Souza Gonçalves, Karin-Soares Cunha, Arley-Silva Junior, Danielle-Castex Conde

**Affiliations:** 1 Ph.D. Postgraduate Program in Pathology, School of Medicine, Universidade Federal Fluminense (UFF), Niterói, RJ, Brazil; 2 M.Sc. Postgraduate Program in Pathology, School of Medicine, Universidade Federal Fluminense (UFF), Niterói, RJ, Brazil; 3 Ph.D. Postgraduation Program in Dentistry, Faculty of Dentistry, Universidade Estácio de Sá, Rio de Janeiro-RJ, Brazil

## Abstract

**Background:**

Assessment of oral epithelial dysplasia is the gold standard for investigating the risk of malignant progression. The World Health Organization (WHO) methods and the binary system have limitations. This study assess the inter- and intra-observer variability of the architectural and cytological criteria and the classification of the presence and degree of epithelial dysplasia in oral lichen planus (OLP) and oral lichenoid lesion (OLL), using both the 2017 WHO criteria and the binary system.

**Material and Methods:**

The sample consisted of 65 biopsies from lesions classified as OLP and OLL according to the American Academy of Oral and Maxillofacial Pathology (AAOMP) criteria. The histological slides were reevaluated by two oral pathologists.

**Results:**

The individual alterations with most inter-observer disagreement were atypical mitotic figures (43.1%), loss of cohesion between epithelial cells (38.5%) and drop shape rete ridges ridges (38.5%). Inter-observer agreement analysis did not show statistically significant agreement regarding the classification of epithelial dysplasia grade by WHO criteria, only regarding the binary system classification (k=0.257; *p*=0.035). Intra-observer agreement analysis by evaluator 1 showed that the classification of epithelial dysplasia grade according to both methods had statistically significant agreement (k=0.546; *p*=0.004, k=0.861; *p*<0.001). Considering evaluator 2, only the evaluation of the WHO system classification showed statistically significant agreement (k=0.593; *p*=0.010).

**Conclusions:**

The evaluation of epithelial dysplasia is subjective and focal changes and inflammatory infiltrate, characteristic of OLP and OLL, can increase the degree of disagreement among evaluators. The binary system presents better inter-observer agreement, while the WHO system presents better intra-observer agreement.

** Key words:**Oral lichen planus, oral lichenoid lesion, oral lichenoid disease, dysplasia, inter-observer variation.

## Introduction

Oral lichen planus (OLP) and oral lichenoid lesion (OLL) represent a heterogeneous group of inflammatory diseases that are characterized by similar clinical manifestations and histopathological features (1). Recently, Aguirre-Urizar *et al*. (2) proposed grouping OLP and OLL under the term “oral lichenoid disease”. However, the American Academy of Oral and Maxillofacial Pathology (AAOMP) believes that distinguishing between the two lesions is important to validate the studies that investigate the biological behavior of these diseases (3). The AAOMP (3) and the World Health Organization (WHO) (4) classify OLL as a potentially malignant disorder (PMD). With regard to OLP, there is still no consensus on its potential for malignancy, and the AAOPM (3) includes the absence of epithelial dysplasia in the set of criteria for its classification. However, the WHO (4) classifies OLP as a PMD.

Oral epithelial dysplasia is a set of architectural and cytological epithelial alterations considered as the gold standard for assessing the risk of progression of premalignant disorders to oral squamous cell carcinoma (5). Many diagnostic methods have been proposed for evaluating and classifying epithelial dysplasia, with the most commonly used being the WHO method (6) and the binary system (7). However, these methods have some limitations due to subjectivity and the lack of guidelines for their classifications.

The aim of this study was to assess the inter- and intra-observer variability of the architectural and cytological criteria and the classification of the presence and degree of epithelial dysplasia in OLP and OLL, using both the 2017 WHO criteria6 and the binary system (7). Furthermore, the study aimed to discuss the difficulties faced by pathologists during the evaluation process of epithelial dysplasia.

## Material and Methods

**Figure 1:**
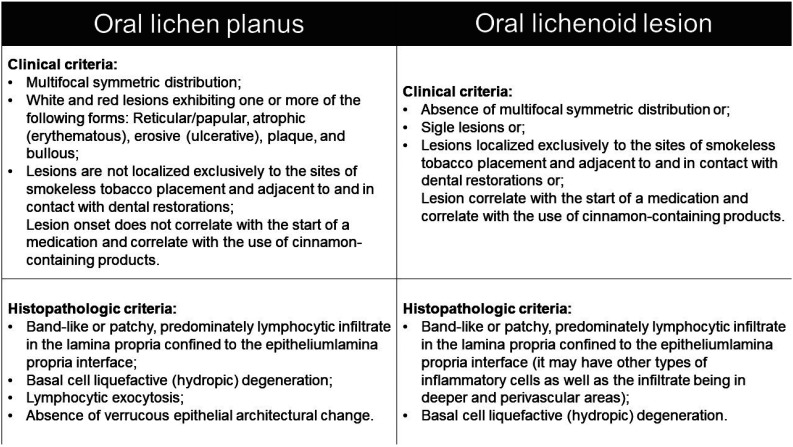
Clinical and histopathologic criteria for the classification of oral lichenoid diseases. Adapted from the American Academy of Oral and Maxillofacial Pathology (3). * For the diagnosis of OLP, all criteria must be included, and for the diagnosis of OLL, only one or more of the criteria must be present.

This study was approved by the Research Ethics Committee of the Antônio Pedro University Hospital of the Fluminense Federal University (APUH-UFF). It is an observational, cross-sectional, and retrospective study. The sample selection was made through a search in the electronic medical records of the Oral Diagnosis Outpatient Clinic of APUH-UFF for patients with oral lichen planus (OLP), oral lichenoid lesion (OLL), oral lichenoid mucositis, and oral lichenoid reaction between the years 2005 and 2019. The lesions were reclassified as OLP and OLL according to criteria of the AAOPM3, with the exception of the criterion of absence of epithelial dysplasia for OLP (Fig. 1).

Lesions with inadequate slides or paraffin blocks for analysis, or those associated with other PMD or candidiasis, were excluded by histopathological analysis through evaluation of stained hematoxylin and eosin (HE) and periodic acid-Schiff (PAS) sections. Cases with candidiasis were excluded due to inflammatory architectural and cytological changes, making the evaluation of epithelial dysplasia difficult (8).

The sample consisted of 65 biopsies (54 of OLP and 11 of OLL) from 56 participants. Seven participants had more than one biopsy from different areas and/or taken at different times.

- Assessment of the presence and grade of epithelial dysplasia

For the analysis of the presence and degree of epithelial dysplasia, histological slides stained with HE were scanned by the computerized system Aperio Technologies Inc.® and evaluated by the Aperio ImageScope® software (Leica Biosystems, Vista, CA, USA). The histological slides of each case was independently reviewed by two experienced oral pathologists (DCC and KSC) to evaluate the individual architectural and cytological criteria, according to the 2017 WHO (6) classification, and the presence or absence of epithelial dysplasia and its degree of severity, according to the 2017 WHO (6) criteria and the binary system (9). The sections were analyzed in their entirety, but the margins of the lesions were not considered to avoid artifacts. The evaluation was performed without information about the original histopathological evaluation and clinical data of the participants.

To assess the presence of epithelial dysplasia, the cytological and architectural criteria recommended by the WHO (6) were investigated. Regarding the degree of epithelial dysplasia according to the WHO (6), mild epithelial dysplasia was considered when changes were restricted to the lower third of the epithelium; moderate dysplasia when such changes extended to the middle third; and severe or intense dysplasia when the upper third was affected by the alterations (6).

For the evaluation of epithelial dysplasia according to the binary system criteria (7), the lesions were categorized as either “low risk” or “high risk”. In this case, the architectural and cytological alterations recommended by the WHO (6) were counted, and lesions that presented at least four architectural alterations and five cytological alterations were classified as high risk. The remaining lesions were considered low risk.

- Evaluation of intra and inter-observer agreement

After histopathological evaluation and classification of epithelial dysplasia, the results generated by the two oral pathologists were compared for inter-observer variability. To evaluate intra-observer agreement, 1/3 of OLP histological slides and 1/3 of OLL histological slides10 were selected and re-evaluated by the same oral pathologists six months after the first evaluation. The sample selection was made randomly using the website Dice throw (11), which generates real-time random numbers by measuring quantum vacuum fluctuations (12).

- Statistical Analysis

The Statistical Package for the Social Sciences® (SPSS), version 23.0, was used for the analyses and the significance level was set at 5% (*p* ≤ 0.05). The intraclass correlation coefficient and the Kappa test were used for the validation and reliability of the evaluation of the presence and severity of epithelial dysplasia. The Landis and Koch (13) evaluation were used to characterize the different ranges of Kappa, with a value less than zero indicating no agreement; between 0.0 and 0.20, indicating slight agreement; between 0.21 and 0.40, indicating fair agreement; 0.41 to 0.60 indicating moderate agreement; 0.61 to 0.80 indicating substantial agreement; and 0.81 to 1.00 indicating almost perfect agreement.

## Results

- Inter-observer variability

Regarding individual evaluation, according to the first evaluation of the 65 biopsies, the histopathological characteristics most frequently observed by evaluator 1 were: increased number and size of nucleoli (n=60/92.3%), loss of polarity of basal layer cells (n=58/89.2%), nuclear hyperchromatism (n=57/87.7%), and abnormal variation in cell shape (n=55/84.6%) (Table 1). The histopathological characteristics most frequently observed by evaluator 2 were: loss of polarity of basal layer cells (n=63/96.9%), increased number and size of nucleoli (n=61/93.8%), nuclear hyperchromatism (n=60/92.3%), and irregular epithelial stratification (n=60/92.3%) (Table 1).

**Table 1:**
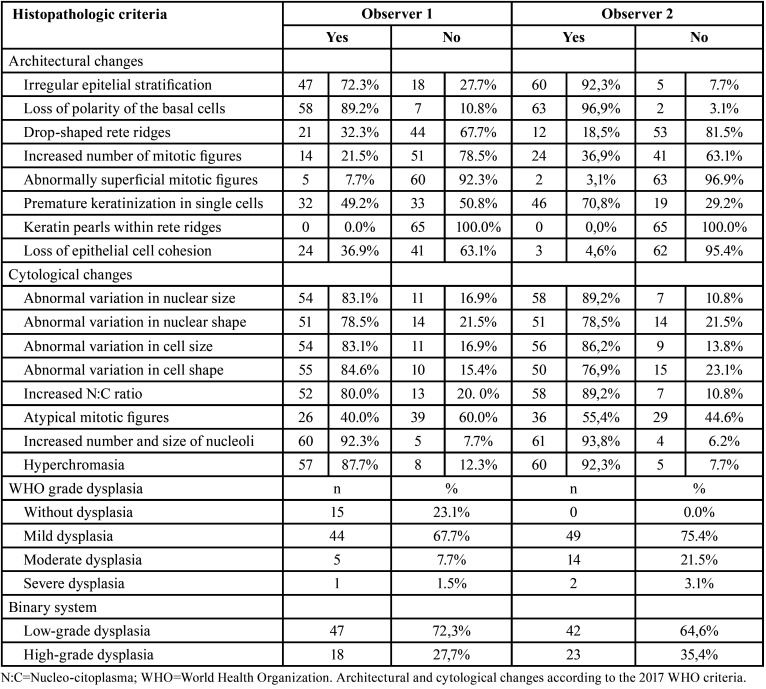
Distribution of classification regarding the presence and absence of individual histopathological criteria and the presence and degree of dysplasia according to each observer.

Regarding inter-observer disagreement of histopathological characteristics for the evaluation of epithelial dysplasia, according to the first evaluation of the 65 biopsies, the changes that most diverged in terms of their presence were: atypical mitotic Figures (n=28/43.1%), loss of cohesion between epithelial cells (n=25/38.5%), and drop shaped rete ridges (n=25/38.5%) (Table 2).

**Table 2:**
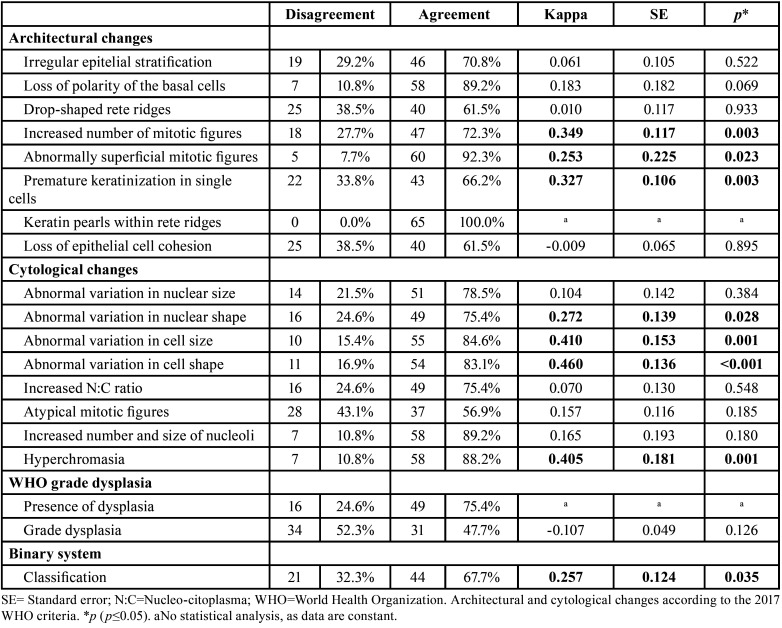
Inter-observer agreement according to individual histopathological criteria for the presence and degree of epithelial dysplasia.

Statistically significant agreements were found in inter-observer variability regarding evaluations of the presence of: increased number of mitotic Figures (k=0.349; *p*=0.003; fair agreement), superficial mitoses (k=0.253; *p*=0.023; fair agreement), premature individual keratinization (k=0.327; *p*=0.003; fair agreement), abnormal variation in nuclear shape (k=0.272; *p*=0.028; fair agreement), abnormal variation in cell size (k=0.410; *p*=0.001; moderate agreement), abnormal variation in cell shape (k=0.460; *p*<0.001; moderate agreement), and nuclear hyperchromatism (k=0.405; *p*=0.001; moderate agreement) (Table 2).

Regarding the presence of epithelial dysplasia according to WHO criteria, evaluator 1 classified 15 (23.1%) biopsies as without epithelial dysplasia and 50 (76.9%) with the presence of epithelial dysplasia, with the majority of biopsies with mild dysplasia (n=44/67.7%). Evaluator 2 did not classify any biopsy as without epithelial dysplasia, and the majority of biopsies were classified as mild epithelial dysplasia (n=49/75.4%) (Table 1).

Regarding classification according to the binary system, evaluator 1 classified 47 (72.3%) biopsies as low risk and 18 (27.7%) biopsies as high risk. Evaluator 2 classified 42 (64.6%) biopsies as low risk and 23 (35.4%) biopsies as high risk (Table 1).

The relationship of inter-observer disagreement in the classification of the presence and degree of epithelial dysplasia according to WHO criteria and the binary system is described in Table 2. No statistically significant agreement was found regarding the classification of the degree of epithelial dysplasia according to WHO criteria. However, there was statistically significant agreement regarding the classification of the binary system (k=0.257; *p*=0.035; fair agreement) (Table 2).

- Intra-observer variability

Evaluator 1 presented four disagreements regarding the presence of epithelial dysplasia and seven regarding the grade of epithelial dysplasia according to WHO criteria (Table 3). Concerning the seven disagreements, it was observed that in three (42.8%) biopsies, the classifications changed from no epithelial dysplasia to mild epithelial dysplasia; in two (28.6%) biopsies, from mild to moderate dysplasia; in one (14.3%) biopsy, from moderate to mild dysplasia; and in another (14.3%) biopsy, from mild dysplasia to no epithelial dysplasia after discussion. It was found that only the individual criteria of increased nucleus-to-cytoplasm ratio and hyperchromatism did not show statistically significant agreement. Abnormal variation in nucleus shape (k=0.861; *p*<0.001) and increased number and size of nucleoli (k=1.000; *p*<0.001) showed almost perfect agreement (Table 3). Both the classification of the grade of epithelial dysplasia according to WHO criteria and the binary system showed statistically significant agreement (k=0.546; *p*=0.004, moderate agreement, and k=0.861; *p*<0.001, almost perfect agreement) (Table 3).

Evaluator 2 presented three disagreements regarding the grade of epithelial dysplasia according to WHO criteria (Table 4), all of which changed from moderate to mild dysplasia after discussion. No statistically significant agreement was found in the individual criteria of loss of polarity of basal cells layer, loss of cohesion between epithelial cells, abnormal variation in nucleus size, abnormal variation in cell shape, and increased nucleus-to-cytoplasm ratio. The evaluation of loss of polarity of basal cells layer (k=1.000; *p*<0.001) and hyperchromatism (k=1.000; *p*<0.001) showed almost perfect agreement. Only the evaluation of the WHO system classification showed statistically significant agreement (k=0.593; *p*=0.010, moderate agreement) (Table 4).

**Table 3:**
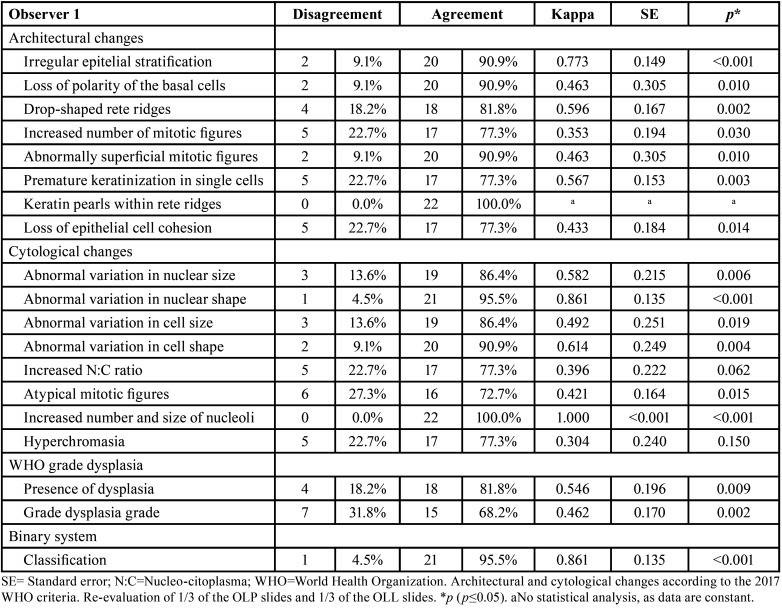
Agreement of the intraobserver assessment by observer 1.

**Table 4:**
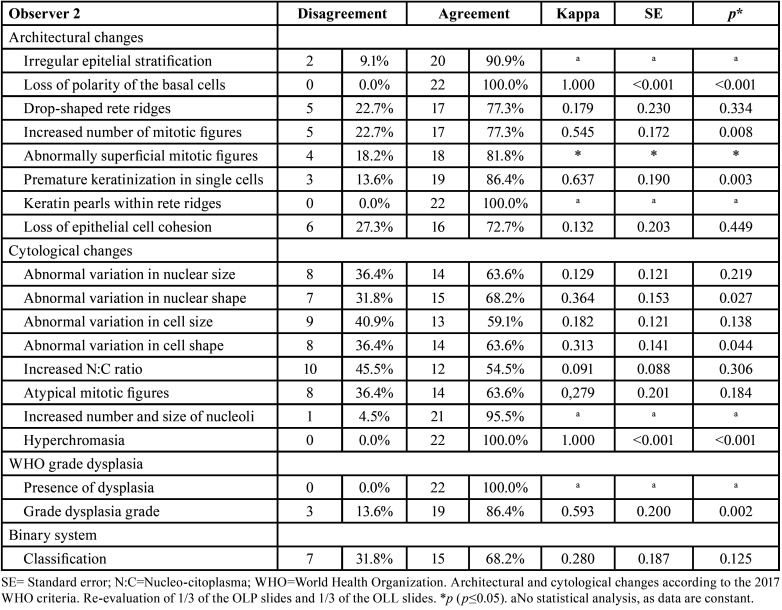
Agreement of the intraobserver assessment by observer 2.

## Discussion

This is the first study to evaluate the intra- and inter-observer agreement of individual architectural and cytological features of epithelial dysplasia in OLP and OLL according to the 2017 WHO criteria and binary system, and to discuss the challenges faced by pathologists in diagnosing epithelial dysplasia and its severity in these lesions.

Applying the “thirds” epithelial classification proposed by the WHO is difficult due to the oral mucosa’s higher degree of heterogeneity, with a wide variation in size, thickness, and complexity of epithelial architecture, making it harder to define the level of “thirds,” particularly in very thin epithelia, in contrast to what occurs in lesions in the cervix, for example (15).

Thus, defining the degree of dysplasia based solely on the number of altered epithelial thirds oversimplifies the complexity of classification, as the presence of cytological atypia in only the basal third may be sufficient for a diagnosis of severe dysplasia, depending on individual characteristics such as bulbous epithelial ridges, and marked cytological atypia (16). Alternatively, lesions with mildly atypical features that extend into the middle third of the epithelium may warrant classification as mild dysplasia (17).

In our study, highest inter-observer agreements among architectural criteria were an increase in the number of mitotic Figures, premature keratinization in single cells, and superficial mitoses. When evaluating cytological criteria, that abnormal variation in cell shape, abnormal variation in cell size, and hyperchromatism showed higher levels of agreement among observers. On the other hand, the most discordant criteria were the loss of cohesion between epithelial cells and drop shaped rete ridges.

The loss of cohesion between epithelial cells was described as an easily recognizable feature (18). However, we observed a limitation in its differentiation from spongiosis caused by inflammation, which is common in oral epithelium, as well as liquefactive degeneration of basal layer cells, characteristics of OLP and OLL. Additionally, an important characteristic found in these lesions is the presence of saw teeth rete ridges (19), which may have made it difficult for evaluators to assess drop shaped projections.

Other studies (20-22) have reported the histopathological characteristics that showed higher and lower agreement in PMD. Kujan *et al*. (21) evaluated 68 cases of epithelial dysplasia by four observers using the WHO (23) criteria of 2005, and demonstrated that increase of mitotic Figures and drop shaped rete ridges had the highest levels of agreement among the observers when assessing architectural changes. Among cytological changes, there was higher agreement in the increase of nucleus size and variation in cell shape. Irregular stratification of the epithelium, loss of polarity, variation in nucleus size, atypical mitosis, and hyperchromatism had the greatest disagreements among observers (21).

Krishnan *et al*. (22), in the evaluation of 63 leukoplakia slides by three observers using the 2005 WHO (23) criteria, reported excellent agreement in the assessment of irregular epithelial stratification, abnormal variation in nucleus size and shape, and increased nucleus-to-cytoplasm ratio. However, they found greater disagreement in the evaluations of superficial mitosis, atypical mitosis, premature keratinization, and increased number of mitotic Figures (22).

In Ranganathan *et al*.’s study (20), using the 2005 WHO (23) criteria, six evaluators analyzed 72 cases of PMD (leukoplakia, erythroplakia, proliferative verrucous leukoplakia, lichen planus, and submucosal fibrosis). There was a higher level of agreement regarding the increase of mitotic Figures, loss of cohesion of epithelial cells, and increased nucleus-to-cytoplasm ratio (20). The greatest disagreements were observed in irregular stratification of the epithelium, loss of polarity of basal layer cells, abnormal variation in nucleus shape, and abnormal variation in cell size.

With these controversial results (20-22), it is difficult to synthesize any guidance on which microscopic features are reported among oral pathologists20. However, despite the increase in the number of mitotic Figures being a subjective analysis and no study indicating how many Figures need to be present, our study and the studies by Kujan *et al*. (21) and Ranganathan *et al*. (20) showed some agreement among evaluators in this analysis. Furthermore, although both evaluators in our study reported difficulties in differentiating Civatte bodies from dyskeratosis in some cases when located in the lower third of the epithelium during individual evaluations, we found considerable inter-observer agreement with respect to this criterion.

The only previous study that evaluated inter-observer agreement of epithelial dysplasia in oral lichenoid diseases, according to the 2017 WHO criteria (6). These authors evaluated 84 cases of proliferative verrucous leukoplakia (PVL) and 28 cases of OLL and found a wide variability in the interpretation of epithelial dysplasia, with low inter-observer reliability among the four examiners (24). The Kappa was classified as very low in the two repeated evaluations among the examiners. Regarding the grading of epithelial dysplasia according to the WHO criteria6, in our study, we also did not find a statistically significant agreement among the evaluators, and the Kappa value was considered very low.

A recent multicenter study (20) evaluated the intra- and inter-observer agreement of six observers who examined PMD (leukoplakia, erythroplakia, proliferative verrucous leukoplakia, lichen planus, and submucosal fibrosis) using the 2005 WHO (23) classification. The study found substantial agreement between two evaluators. However, the other observers had poor to fair (20).

Speight *et al*. (14) highlight that the highest levels of disagreement are found when categorizing low-grade lesions due to the fact that these lesions present more subtle changes, which can be observed in reactive lesions as a result of an inflammatory infiltrate (14). Therefore, we believe that the low values of inter-observer agreement in our study may also be related to the fact that the majority of our sample of OLP and OLL presented subtle cytological and architectural changes, as well as the intense inflammatory infiltrate, which is characteristic of these lesions.

Regarding intra-observer agreement, the study by Zohdy *et al*. (24) conducted in oral lichenoid diseases reported a slight improvement in intra-observer reliability during the evaluation of the epithelial dysplasia grading by the WHO system (6), which varied among examiners.

In our study, we found better intra-observer agreement between the two evaluators. The evaluator 1 showed higher agreement in evaluating cytological criteria, while evaluator 2 showed higher agreement in evaluating architectural criteria. Regarding architectural criteria, we found that the highest agreements were for loss of polarity of basal cells layer, increased number of mitotic Figures, and individual premature keratinization of the cell. On the other hand, abnormal variation in nucleus shape and abnormal variation in cell shape were the cytological criteria with the highest agreements.

In an attempt to minimize inter- and intra-observer disagreements, many authors (15,20) recommend the use of the binary system (7), as they believe this approach is standardized and could overcome subjectivity in reporting epithelial dysplasia (20). However, in our study, despite finding better inter-observer agreement with the binary system7 compared to the WHO system (6), evaluator 2 showed better reliability in assessing the classification of epithelial dysplasia according to the 2017 WHO criteria.

These results reinforce that grading of epithelial dysplasia is therefore subjective, lacks reproducibility, and may be influenced by evaluators’ experience, fatigue, and emotional factors (25). It has been shown that more anxious individuals tend to behave more negatively when making professional decisions (26), suggesting potential consequences in microscopic interpretation.

Despite their limitations, the evaluation of epithelial dysplasia offers the pathologist the best opportunity to convey the overall risk of malignancy to the clinician (14). Additionally, high concordance of a method does not indicate that it is the most correct for use, only that it is reproducible. Thus, we emphasize the importance of defining guidelines to improve the interpretation of criteria involving the evaluation of epithelial dysplasia and reduce inter-observer variability. We also highlight the need for studies that seek to correlate the classification of epithelial dysplasia in both systems with the risk of malignant transformation.

This study has some methodological limitations due to its retrospective design. In addition, despite the pathologists having training in Pathology from the same institution and working together for years, there was no prior standardization before the evaluations. Furthermore, it has been questioned in the literature whether the Kappa statistic, used to measure reproducibility in a dichotomous decision model in pathology, is the most appropriate (27), as it is believed that these verbal descriptors of reproducibility can be arbitrary (15). However, it is still the most widely used in the majority studies to date (20-22,24).

The evaluation of both the presence of individual criteria and the classification of the presence and degree of epithelial dysplasia is subjective and dependent on the individual experience of the evaluators. The most discordant criteria among evaluators in the assessment of OLP and OLL are the evaluation of loss of cohesion of the epithelial cells and drop shaped rete ridges. The subtle and focal changes and the inflammatory infiltrate, characteristic of these lesions, can increase the degree of disagreement between evaluators. The binary system shows better inter-observer agreement, while the 2017 WHO system shows better intra-observer agreement.
